# Factor correction as a tool to eliminate between-session variation in replicate experiments: application to molecular biology and retrovirology

**DOI:** 10.1186/1742-4690-3-2

**Published:** 2006-01-06

**Authors:** Jan M Ruijter, Helene H Thygesen, Onard JLM Schoneveld, Atze T Das, Ben Berkhout, Wouter H Lamers

**Affiliations:** 1 Department of Anatomy and Embryology, Academic Medical Centre, Meibergdreef 15, 1105 AZ Amsterdam, The Netherlands; 2 Department of Clinical Epidemiology and Biostatistics, Meibergdreef 15, 1105 AZ Amsterdam, The Netherlands; 3 AMC Liver Center, University of Amsterdam, Meibergdreef 69-71, 1105 BK, Amsterdam, The Netherlands; 4 Laboratory of Signal Transduction, National Institute of Environmental Health Sciences, National Institutes of Health, Research Triangle Park, NC, USA; 5 Department of Human Retrovirology, Academic Medical Centre, Meibergdreef 15, 1105 AZ Amsterdam, The Netherlands

## Abstract

**Background:**

In experimental biology, including retrovirology and molecular biology, replicate measurement sessions very often show similar proportional differences between experimental conditions, but different absolute values, even though the measurements were presumably carried out under identical circumstances. Although statistical programs enable the analysis of condition effects despite this replication error, this approach is hardly ever used for this purpose. On the contrary, most researchers deal with such between-session variation by normalisation or standardisation of the data. In normalisation all values in a session are divided by the observed value of the 'control' condition, whereas in standardisation, the sessions' means and standard deviations are used to correct the data. Normalisation, however, adds variation because the control value is not without error, while standardisation is biased if the data set is incomplete.

**Results:**

In most cases, between-session variation is multiplicative and can, therefore, be removed by division of the data in each session with a session-specific correction factor. Assuming one level of multiplicative between-session error, unbiased session factors can be calculated from all available data through the generation of a between-session ratio matrix. Alternatively, these factors can be estimated with a maximum likelihood approach. The effectiveness of this correction method, dubbed "factor correction", is demonstrated with examples from the field of molecular biology and retrovirology. Especially when not all conditions are included in every measurement session, factor correction results in smaller residual error than normalisation and standardisation and therefore allows the detection of smaller treatment differences. Factor correction was implemented into an easy-to-use computer program that is available on request at: biolab-services@amc.uva.nl?subject=factor.

**Conclusion:**

Factor correction is an effective and efficient way to deal with between-session variation in multi-session experiments.

## Background

**Figure 1:**
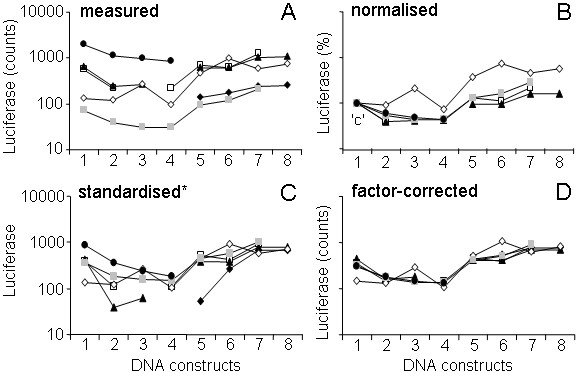
**Comparison of normalisation, standardisation and factor correction**. DNA constructs containing different enhancer, promoter, and intron sequences from the rat glutamine synthetase gene coupled to the firefly luciferase reporter gene were transfected into FTO-2B cells. Luciferase activity was measured 64 hours after transfection [1]. This plot shows the activity of 8 different DNA constructs (= conditions) measured in 6 independent measurement sessions (◆ □ ▲ ◇  ●). **A**: Original measurements, plotted on a logarithmic Y-axis. The approximately parallel lines connecting the results from each session indicate that most of the variation between the sessions is multiplicative. **B**: Data after normalisation, using condition 1 as 'control' (one session [◆] did not include condition 1 and had to be dropped). Note that the variation in the control condition ('c') is lost. **C**: Data after standardisation. Note that a linear transformation of the standardised values (standardised* = 410 + 305 × standardised) was required to enable this logarithmic plot. **D**: Data after applying factor correction. The minimal remaining distance between the lines indicates that factor correction is most effective in removing the multiplicative between-session variation.

**Figure 2:**
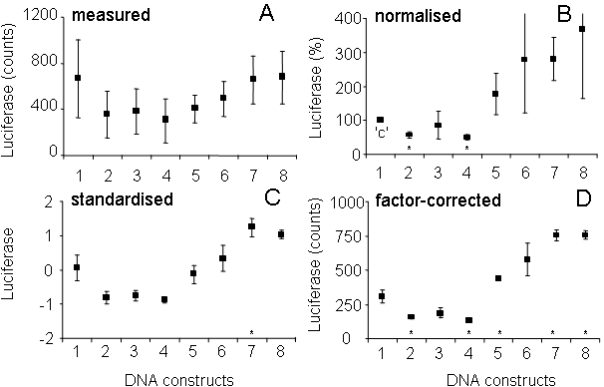
**Comparison of normalisation, standardisation and factor correction**. Mean (and SEM) of the data of the molecular-biology data set from Figure 1 **A**: original data. **B**: normalised data. **C**: standardised data. **D**: data after factor correction. Note that normalisation, standardisation, and factor correction reduce the variation within each condition. However, normalisation (**B**) leads to loss of variance in the control condition ('c') and to added variation in the other conditions. Standardisation (**C**) of this incomplete data set leads to increased variation, compared to factor correction, in some conditions. With factor correction (**D**) all conditions retain their statistical variance, which is generally smaller than after normalisation and standardisation. An asterisk indicates a statistically significant difference between the DNA construct and construct 1 (t-test; P < 0.05). Note that the number of observations per construct in these comparisons ranges from 2 to 5.

In experimental biology, including retrovirology and molecular biology, replicating a series of measurements under presumably identical circumstances often leads to results that show the same proportional differences between experimental conditions, but very different absolute values within each of the conditions. As an example Figure 1A shows data from a multi-session experiment in which multiple promoter-luciferase-reporter constructs were transfected into hepatoma cells. Luciferase activity was quantified two days after transfection [1]. Although the different constructs demonstrate a similar pattern of luciferase activity in each of the sessions, the activity for some of the constructs can vary up to 30-fold in different sessions. This between-session variation results from small, but systematic, differences in e.g. cell density, substrate and reagent concentration, reaction temperature and exposure time, which all can be shown to proportionally increase or decrease the outcome of all biological measurements in a session [2]. The between-session variation can therefore be modelled as a multiplicative factor working on the data in each session. As exemplified in Figure 1A, the between-session variation can be very large and may conceal differences between the activities of the different constructs. A pair-wise comparison of each of the DNA constructs with construct 1 indeed revealed no statistically significant differences in the measured data (Fig. 2A; t-test, all P > 0.4). One way to test whether the activity of constructs differs despite this confounding between-session variation is to apply analysis of variance (ANOVA). However, even though this method is available in statistical programs, ANOVA is hardly ever used for this purpose in biochemistry, virology or molecular biology because these programs are elaborate and hard to use for the non-expert. In practice, most researchers use their own 'normalisation' method, which is often not validated and seldom mentioned in the methods section of the paper. The importance of using good and reliable statistical methods was recently discussed in detail for the field of virology [3] but obviously holds for all disciplines of experimental biology and medicine [4]. However, in these papers the handling of this between-session variation is not discussed. The current paper intends to bridge the gap between statistical theory and laboratory practice with respect to the removal of between-session variation.

The most popular methods used to remove between-session variation in bio-medical research are "normalisation" and "standardisation" [5]. In normalisation, a "control" condition is defined and per session all measured values (Y_ni_) are divided by the control value in the session (Eq. 1, with session n, condition i and control condition 1).



Thus a single control condition is chosen to serve as a correction factor (100/Y_n1 _in Eq. 1). Figure 1B shows the data from Figure 1A when normalisation, using DNA construct 1 as control, is applied. Since DNA construct 1 was lost in one session (◆) normalisation led to the loss of this entire session. Normalisation does remove some between-session variation but because the control condition itself carries biological error, this can lead to an increased variation. The variation for constructs 6 and 8, i.e., is much larger after normalisation compared to the original data (compare Figs. 2A and 2B). Another drawback of normalisation is that it generates a control condition without variation. Since parametric statistical tests for the comparison of two or more conditions assume an equal variance in all conditions [6] these tests can no longer be used. Also most nonparametric tests are no longer applicable, because they require similar distributions in all conditions [7].

In standardisation [5], each value per session is transformed into a standard value by subtracting the session mean () and dividing the result by the session standard deviation (SD_n_, Eq. 2).



Because the session mean after standardisation becomes zero for each session, standardisation removes between-session variation (Fig. 1C). However, the original measurement scale is lost and the overall mean becomes zero. Furthermore, if not all conditions are present in every session, the session mean and standard deviation will be biased. Because the standard deviation serves as multiplicative correction factor, this bias can result in added variability between sessions (as observed for the sessions indicated with triangles and filled diamonds in Fig. 1C). Standardisation can, therefore, only be used effectively when the data set is complete, that is, when all conditions are present in every session.

As mentioned above, the between session variation is due to multiplicative session factors. When known, these factors can be used to correct the data. As was demonstrated in the previous paragraphs, normalisation and standardisation both use correction factors that can lead to ineffective correction or even to an increased variation within conditions. For a correction method to be effective, the correction factors should be based on all available observations in the session and the estimation of these factors should not be affected by incomplete data sets. This paper describes such a correction method, dubbed "factor correction" and introduces two approaches to estimate correction factors. In the first, "ratio", approach the variation in the data set is assumed to be restricted to the condition effects whereas in the second, "maximum likelihood" approach part of the variation may result from variation among the factors affecting the individual measurement in each session. Both approaches turn out to result in very similar correction factors. Their use and effectiveness are illustrated using data sets from molecular biology and retrovirology.

## Results

### Mixed additive and multiplicative model

In the molecular-biology data set plotted in Figure 1A, the different DNA constructs represent the experimental conditions. Data from transfection experiments carried out on different days are the measurement sessions. The multiplicative nature of the between-session variation in this data set is apparent from the fact that the lines connecting the data points in each session run approximately parallel in a logarithmic plot of the data (Fig. 1A). In a multi-session experiment with such a multiplicative between-session variation, the observations can be described with a mixed additive and multiplicative model (Eq. 3).

Y_ni _= F_n _× (Y_mean _+ E_i _+ error_ni_)     (Eq. 3)

The additive part of this model, between parenthesis, states that the result of a measurement Y in condition i is the sum of the population mean (Y_mean_), the effect of condition i (E_i_), and an experimental error. Note that 'effect' in the sense used here does not represent the difference between a control and an experimental condition, but stands for the effect of each condition relative to the population mean. Therefore, the sum of the condition effects is 0 (). In this model the biological error is normally distributed with mean 0 and standard deviation *σ*. This biological error reflects the variance *within *a condition, whereas the condition effects reflect the differences *between *conditions [6]. For each session n, the additive part of the observation is multiplied by session factor F_n_. The product of the session factors equals 1 (), which insures that the mean of Y_ni _is still equal to the overall Y_mean_.

The session factors can be estimated from all available data in the multi-session data set with two different approaches: calculation of a between-session ratio matrix (Ratio approach) or a maximum likelihood approach.

### Estimation of the session factors with the Ratio approach

To estimate the session factors with the Ratio approach for each pair of sessions, a between-session ratio is calculated (Eq. 4). For e.g. session 5 and 6, and condition i, this ratio is:



In such a between-session ratio, the normally distributed additive parts of the multi-session model (Y_mean _+ E_i _+ error_ni_), have the same mean and standard deviation, and hence a ratio of 1. The error of such a ratio of normally distributed variables has a Cauchy distribution [8], which implies that, strictly speaking, its mean does not exist. However, the Cauchy distribution has a symmetrical clock shape centred on zero, has a median of zero [8] and, with a more general definition of integration, its mean can also be considered to be zero [9]. Therefore, on average, the error in the last term of Eq. 4 is zero and the term cancels out which makes the between-session ratio an unbiased estimate of the ratio of two session factors. When two sessions have more than one condition in common, a between-session ratio is calculated for each matching pair of conditions. Because we are dealing with multiplicative effects, the geometric mean of these ratios [10] is used in the between-session ratio matrix.

In the example data set (Fig. 1A), sessions 1 and session 6 have no conditions in common and, therefore, a between-session ratio cannot be directly calculated for this pair of sessions. To be able to calculate proper session factors without the loss of data sets like sessions 1 and 6, missing between-session ratios have to be substituted. It is possible to calculate a substitution for a missing ratio in column j and row i (R_j/i_) from a known ratio in that column (e.g. R_j/n_) and two other ratios from these two rows in another column (R_k/i _and R_k/n_). A substitute value for the missing ratio R_j/i _is then calculated as R_j/i _= R_j/n _× R_k/i_/R_k/n_. If such a substitute is computed for all possible R_j/n_, R_k/i_, and R_k/n _the geometric mean of all values will be the best estimate of the missing ratio R_j/i_.

Because the product of all session factors in the multi-session model equals 1, the geometric mean of column i in this between-session ratio matrix is an estimate of the correction factor for session i:



The between-session variation in the original data set can now be removed by dividing each measured value by the corresponding session factor (Eq. 6):



The corrected data are shown in Fig. 1D.

### Estimation of session factors with the maximum likelihood approach

In the above mixed additive and multiplicative model the error term is normally distributed with a standard deviation *σ*. When we define  = *σ*·*F*_*n *_and  = *Y*_*i*_/*σ *with Y_i _as the mean value per condition (Y_i _= Y_mean _+ E_i_; see Eq. 3) the model can be rewritten as *Y*_*ni *_=  ( + *error*_*ni*_/*σ*), and  can then be shown to be normally distributed with mean 0 and standard deviation 1. Based on this form of the model, the likelihood of the observed set of Y_ni _is given by Eq. 7



which is the chance of finding each individual observation Y_ni _given F_n _and Y_i_, multiplied (Π) for all observations.

If this likelihood function is maximal for  = Y_i,max_,  = F_n,max_, then Y_i,max _and F_n,max _are found when the first derivatives in Y and F of the log of this likelihood function equal 0. The estimation equations for Y_i _and F_n _are not independent of each other and, therefore, an iterative procedure is required to estimate the sets of Y_i,max _and F_n,max _parameters.

This maximum likelihood approach results in a set of session factors (F_n_) as well as estimates of condition means (Y_i_). For both sets of parameters the maximum likelihood approach also estimates standard errors that can be used to compare factors and condition means among each other. Note that in this approach part of the variation in the data set is attributed to a variation in factor effect within a session. This is in contrast to the above ratio approach in which the factors are assumed to be fixed.

Table 1 gives an example of the calculation of session factors using each of the methods on a simulated data set. The session factors of both methods, as well as the condition means resulting from the maximum likelihood method, are very close to the values used in the simulation. The session factors resulting from the ratio approach fall within the confidence interval of those estimated with the maximum likelihood method (t-test; all P > 0.6). A computer program that performs factor correction with both approaches is available on request at: biolab-services@amc.uva.nl?subject=factor.

**Table 1:** Results of the application of both methods for estimation of session factors on a simulated data set. A multi-session experiment with 5 sessions and 5 conditions was simulated with 5 observations per combination of session and condition. Each condition was measured in 4 different sessions. In simulating data, the overall mean was set to 100 and the standard deviation was set to 10. Factors and condition effects are given in the table. The estimated session factors are all close to the factors used in the simulation for both methods and the factors estimated with the ratio method are well within the variance of those estimated with the maximum likelihood approach. The condition means estimated with the maximum likelihood method are close to the values used in the simulation.

Ymean	sd	n	se	
100	10	20	2.24	

	simulated	ratio observed	max. likelih. observed	
session	factor	factor	factor	se

1	0.1	0.101	0.101	0.002
2	0.2	0.188	0.188	0.004
3	1	1.065	1.054	0.021
4	5	4.913	4.979	0.093
5	10	10.05	10.02	0.185

	simulated		observed	
condition	effect		mean	se

A	-50		51.7	2.14
B	-20		78.6	2.14
C	0		101.7	2.15
D	20		119.4	2.15
E	50		151.4	2.16

### Application of factor correction to molecular-biology data set

The result of normalisation and standardisation of the incomplete data set from Figure 1A are shown in Figures 1B and 1C and were discussed above. The result of factor correction (ratio approach) is plotted in Figure 1D. The factors estimated by maximum likelihood result in a graph that is indistinguishable. The reduced distance between the session lines in Figure 1D, compared to Figure 1A, shows that the multiplicative between-session variation has been removed successfully. This is also shown by the reduced variation within the conditions after factor correction (compare Fig. 2A and Fig. 2D). The remaining difference between the session lines (Fig. 1D) reflects the non-multiplicative component of the variation, which represents the error component in the multi-session model (Eq. 3). Compared to normalisation (Figs. 1B and 2B) and standardisation (Figs. 1C and 2C) the within-condition variation after factor correction is clearly reduced, demonstrating that factor correction is more effective in the removal of between-session variation. When the factor-corrected data are used to test the differences between each of the DNA constructs and construct 1, only constructs 3 and 6 are not significantly different (t-test; P = 0.095 and P = 0.071, respectively; Fig. 2D). The same test applied to normalised and standardised data reveals that only 2 and 1 DNA constructs, respectively, that differ significantly from construct 1 (asterisks in Figs. 2B and 2C). These results demonstrate that the power of the statistical comparison clearly increases after factor correction.

### Application of factor correction to retrovirology data set

**Figure 3:**
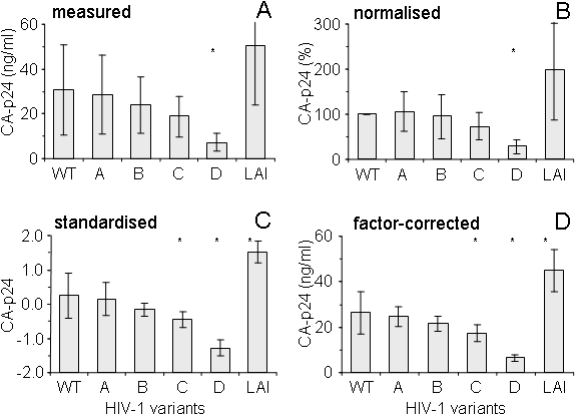
**Virus production of HIV-1 variants**. The HIV-1 molecular clone LAI and derivatives with a modified mechanism of transcription regulation [13] and variation in the viral Tat gene were transfected into C33A cells. Virus production was measured at two days after transfection. The experiment was repeated seven times. **A**: mean values with standard deviation of observed data. **B**: normalisation of the data with the WT construct set at 100% in each session. **C**: corrected data after standardisation. **D**: data after removal of between-session variation with factor correction. WT: HIV-rtTA construct with wild-type Tat gene; A-D: HIV-rtTA variants with mutated Tat genes (to be described elsewhere); LAI: HIV-LAI proviral clone with unmodified mechanism of transcription regulation. An asterisk indicates a statistically significant difference between the virus variant and WT (t-test; P < 0.05). The number of observations per variant is 8.

We also demonstrate the effectiveness of factor correction with a data set that originates from the field of HIV-1 virology. When testing different HIV-1 variants, it is standard practice to construct infectious proviral clones and to test their capacity for gene expression and virus production upon transfection of cells. As an example, Figure 3A shows an experiment in which 6 HIV-1 variants were transfected into cells and virus production was monitored by measuring the viral structural protein CA-p24 in the culture supernatant at two days after transfection. The mean and standard deviation of the data from seven measuring sessions are shown. This HIV-1 virology data set was a complete set. The between-session variation, which is due to variation in transfection efficiency and other experimental variation, clearly results in relatively large standard deviations. Normalisation of the data reduces the standard deviation, but the variation in the 'control' sample is lost (Fig. 3B). Because the data set is complete, the correction by standardisation is effective in removing the between-session variation but leads to loss of the original measurement scale (Fig. 3C). Applying factor correction to eliminate the between-session variation also reduces the standard deviation for each virus but preserves the original scale. A series of t-tests between the wild type and each of the other HIV-1 variants showed that according to the measured data (Fig. 3A) only variant D differed significantly from wild type (P = 0.022). After factor correction (Fig. 3D) significant differences from wild type could be observed for variants C, D and LAI (P-values: 0.033, 0.001 and 0.003, respectively).

## Discussion

This paper describes factor correction as an effective method to remove between-session variation from multi-session experiments. Using data sets from the fields of molecular biology and retrovirology, we demonstrate that factor correction effectively eliminates between-session variation in both complete and incomplete data sets. The corrected data set can be used reliably for statistical testing of differences between conditions, because the statistical error is not affected by factor correction. Moreover, the scale of the factor-corrected values can be considered to represent the original measurement scale.

Similar to normalisation and standardisation, factor correction is based on a multiplicative model for the variation observed in such multi-session experiments (Eq. 3). After normalisation, standardisation, and factor correction, the pattern of between-condition differences is very similar (Figs. 2 and 3). However, in normalisation, the control condition has lost its variance and the variance of all other conditions is larger than when factor correction is applied (cf. Figs. 2B and 2D, 3B and 3D). In other words, the variation that is lost in the control condition has been added to the other conditions. This is because the users of normalisation implicitly, but unjustifiably, assume that the control condition is error-free. Because the HIV-1 virology data set was complete the standardised and factor-corrected data set are very similar (cf. Figs. 3C and 3D). However, when standardisation is applied to an incomplete data set, both the session mean and the session standard deviation are not corrected for missing conditions, which may increase the variation for some conditions. The variation that is observed for e.g. constructs 2 and 5 in the molecular-biology data set is clearly larger after standardisation than after factor correction (cf. Figs. 2C and 2D). In factor correction, all available data are equally weighted to estimate session factors, which allows its use for incomplete data sets.

An alternative method to estimate the multiplicative factors in the mixed additive and multiplicative model is the use of two-way ANOVA after a logarithmic transformation of the data which converts the multiplicative session factor into an additive component. The application of two-way ANOVA without interaction between session and condition then results in a log-factor per session. Note that the condition effects that result from this two-way ANOVA are calculated as multiplicative effects and this will cause the factor estimates to differ marginally from those calculated either with the ratio approach or by maximum likelihood estimation (data not shown).

The two methods to estimate session factors described in this paper give slightly different results because the maximum likelihood approach assigns part of the variation to the estimated session factors. The ratio approach can be seen as a special case, in which the user assumes that the multiplicative factor is the same for every measurement in a session. Therefore, the maximum likelihood method is the more generally applicable of the two methods. In this paper the equations for the maximum likelihood approach have been developed for a one-way experimental design. Because the focus of this paper is to present an alternative for the unsound normalization often applied in the laboratory, we did not pursue the maximum likelihood estimation of session factors for more complex experimental designs. However, the current design enables the calculation of session factors as if the design is one-way and the application of these factors. The resulting factor-corrected data can then be used in a statistical package for further analysis.

When factor correction is used, sessions no longer have to be discarded because of loss of some data points in the laboratory procedure. Moreover, factor correction enables the correction of multi-session data sets that are necessarily incomplete because more conditions have to be tested than can be measured per session. Furthermore, because the control condition is no longer required in each session, resources can be used more efficiently. The smaller within-condition error after application of factor correction, as compared to normalisation and standardisation, increases the power of the statistical tests of biological hypotheses and reduces the required number of observations.

## Conclusion

We present factor correction as an effective and efficient method to eliminate between-session variation in multi-session experiments. The method was implemented in an easy-to-use computer program that is available on request at: biolab-services@amc.uva.nl?subject=factor. Factor correction helps experimental biologists to find the needle of biologically relevant information in the haystack of between-session variation.

## Methods

### Molecular-biology data set

The aim of the study from which this data set is derived was to examine the transcriptional activity of different combinations of enhancer, promoter and first intron elements of the rat Glutamine Synthetase (GS) gene [1]. To this end, DNA constructs containing different enhancer-promoter-intron sequences in front of the luciferase reporter gene were transfected into rat FTO-2B hepatoma cells by electroporation. Cells were co-transfected with a chloramphenicol acetyltransferase expression plasmid (pRSVcat). Sixteen hours after transfection the medium was refreshed and another 48 hours later the cells were harvested and tested for luciferase and CAT activity. The activity of the tested DNA construct was expressed as the ratio between the luciferase activity and the CAT activity.

### HIV-1-virology data set

HIV-1 constructs with a modified mechanism of transcription regulation [13] and variation in the viral Tat gene (to be described elsewhere) were transfected into human C33A cervix carcinoma cells as previously described [14]. Virus production was measured by CA-p24 ELISA on culture supernatant samples two days after transfection. The experiment was repeated seven times.

## Competing interests

The author(s) declare that they have no competing interests.

## Authors' contributions

WL conceived the idea of using between-session ratios to correct for between-session variation in incomplete data sets and JR worked out the mixed additive and multiplicative data model for this purpose. HT developed the maximum likelihood method to estimate session factors. JR and HT implemented both methods in a computer program and JR drafted the manuscript. OS, AD and BB contributed by supplying the sample data sets and testing of the procedure in transfection experiments. All authors read, corrected and approved the final manuscript.
